# Protection from SARS-CoV-2 Variants by MVAs expressing matched or mismatched S administered intranasally to mice

**DOI:** 10.1038/s41541-023-00645-7

**Published:** 2023-03-27

**Authors:** Catherine A. Cotter, Jeffrey L. Americo, Patricia L. Earl, Bernard Moss

**Affiliations:** Laboratory of Viral Diseases, National Institute of Allergy and Infectious Diseases, National Institutes of Health, Bethesda, MD USA

**Keywords:** Live attenuated vaccines, Preclinical research

## Abstract

SARS-CoV-2 vaccines prevent severe disease but are less efficient in averting infection and transmission of variant strains, making it imperative to explore ways of enhancing protection. Use of inbred mice expressing the human SARS-CoV-2 receptor facilitates such investigations. We employed recombinant MVAs (rMVAs) expressing modified S of several SARS-CoV-2 strains and compared their ability to neutralize variants, bind S proteins and protect K18-hACE2 mice against SARS-CoV-2 challenge when administered intramuscularly or intranasally. The rMVAs expressing Wuhan, Beta and Delta S induced substantial cross neutralizing activities to each other but very low neutralization of Omicron; while rMVA expressing Omicon S induced neutralizing antibody predominanly to Omicron. In mice primed and boosted with rMVA expressing the Wuhan S, neutralizing antibodies to Wuhan increased after one immunization with rMVA expressing Omicron S due to original antigenic sin, but substantial neutralizing antibody to Omicron required a second immunization. Nevertheless, monovalent vaccines with S mismatched to the challenge virus still protected against severe disease and reduced the amounts of virus and subgenomic RNAs in the lungs and nasal turbinates, though not as well as vaccines with matched S. Passive transfer of Wuhan immune serum with Omicron S binding but undetectable neutralizing activity reduced infection of the l–ungs by Omicron suggesting additional effector functions. Notably, there was less infectious virus and viral subgenomic RNAs in the nasal turbinates and lungs when the rMVAs were administered intranasally rather than intramuscularly and this held true for vaccines that were matched or mismatched to the challenge strain of SARS-CoV-2.

## Introduction

The speed with which safe and efficacious SARS-CoV-2 vaccines were developed was a remarkable achievement. Clinical trials indicated that the mRNA vaccines were 94 to 95% effective in preventing confirmed cases of COVID-19^1,2^ and adenovirus-based vaccines were about 74% effective^3,4^. Those and most other vaccines are based on the spike (S) protein, which mediates entry of the virus into cells. Initially, it was considered that the proof-reading mechanism employed by coronaviruses would greatly retard the development of escape mutants^5^. However, successive waves of variants and subvariants appeared with mutations in S including the receptor binding domain (RBD) and some such as Beta and Omicron exhibited resistance to antibodies elicited by ancestor strains^6^. Nevertheless, boosting with the original vaccines reduce serious disease, though they appear less effective in preventing infection and transmission^7^. Updated SARS-CoV-2 mRNA vaccines are based on expression of two S proteins: one from an ancestor and the other from Omicron BA.1^8^. Considerations for the future are whether vaccines need to be continually updated with S variants and whether intranasal (IN) or aerosol delivery would prevent infection and transmission better than intramuscular (IM) vaccination.

Recombinant poxvirus platforms are valuable for identifying targets of humoral and cellular immunity, have been developed into numerous veterinary vaccines and are undergoing clinical evaluation for vaccines against many pathogens including SARS-CoV-2 as well as for cancer^9–11^. We and others described animal studies supporting use of the host-range restricted vaccinia virus Ankara (MVA) as an alternative vector for COVID-19 vaccines^12–16^. Recent animal studies demonstrated advantages of IN delivery of recombinant MVAs (rMVAs) expressing S^17–19^. In those studies anti-SARS-CoV-2 IgA and IgG as well as specific T cells were detected in the lungs of IN vaccinated mice and virus was diminished in the upper and lower respiratory tracts following challenge of K18-hACE2 mice or hamsters with SARS-CoV-2. Here we describe the construction and immunogenicity of rMVAs expressing the S proteins of several variant SARS-CoV-2 strains. The neutralizing and S binding activities of sera following matched and mismatched rMVA boosts were determined as well as protection of K18-hACE2 mice vaccinated IN and IM and challenged IN with SARS-CoV-2 variants. Vaccines that produced low neutralizing activities to mismatched SARS-CoV-2 variants still provided durable protection against weight loss and death, but vaccines matched to the challenge virus elicited higher neutralizing activities and were more effective. For both matched and mismatched immunizations, the IN route was better than IM at reducing virus infection of the upper and lower respiratory tracts. In mice previously primed and boosted with rMVA expressing ancestral Wuhan S, antibodies to the Wuhan S increased after one immunization with rMVA expressing Omicron S due to original antigenic sin, but substantial neutralizing antibody to Omicron required a second immunization.

## Results

### Relative virulence of SARS-CoV-2 variants in the K18-hACE2 mouse model

We previously reported that serum from mice vaccinated IM with an rMVA expressing the spike protein of the ancestor Wuhan strain of SARS-CoV-2, that was triply modified by stabilization of the prefusion structure by proline substitutions, inactivation of the furin cleavage site and deletion of the endoplasmic retention signal (rMVA-W), neutralized recombinant vesicular stomatitis virus (rVSV) pseudoviruses expressing divergent S proteins to varying degrees^18^. This cross-reactivity led us to evaluate the ability of rMVA-W and other rMVAs expressing variant S proteins to protect K18-hACE2 mice against challenge with SARS-CoV-2 variants. In order to undertake protection studies, we first compared the relative virulence of four SARS-CoV-2 variants, CoV-Washington (W) that has S identical to Wuhan, CoV-Beta (B), CoV-Delta (D), and CoV-Omicron BA.1.1 (O) in this mouse model system. Amino acid differences between the S proteins of the ancestral and later variants used for challenge are shown in Supplementary Fig. 1 (Also shown are altered S sequences in the rMVA vaccines and pseudoviruses, which will become relevant later). After IN infection of the hACE2 mice with CoV-W, -B and -D, weight loss was detected with a dose of 10^2^ TCID_50_ (Supplementary Fig. 2a–c) and 100% death occurred with 10^4^ TCID_50_ (Supplementary Fig. 2d–f). CoV-O was less virulent than other strains, as also shown by others^20,21^, with only two of five mice succumbing on day 7 to a dose of 5 × 10^4^ (Supplementary Fig. 2g). For a further comparison, the hACE2 mice were inoculated IN with varying doses of CoV-W or -O and the amounts of virus in the lungs and nasal turbinates determined. The lungs of CoV-W-infected mice contained 100- to 1000-fold more virus than the lungs of CoV-O-inoculated mice on day 2 (Supplementary Fig. 2h). At that early time, virus was detected in the nasal turbinates only of mice inoculated with CoV-W (Supplementary Fig. 2i). In subsequent challenge experiments, a dose of 5 × 10^4^ TCID_50_ (highest possible dose due to titer) was used for CoV-O and 10^4^ TCID_50_ for the others.

### Protective immunity to SARS-CoV-2 variants following IM vaccination with rMVA-W

**Fig. 1:**
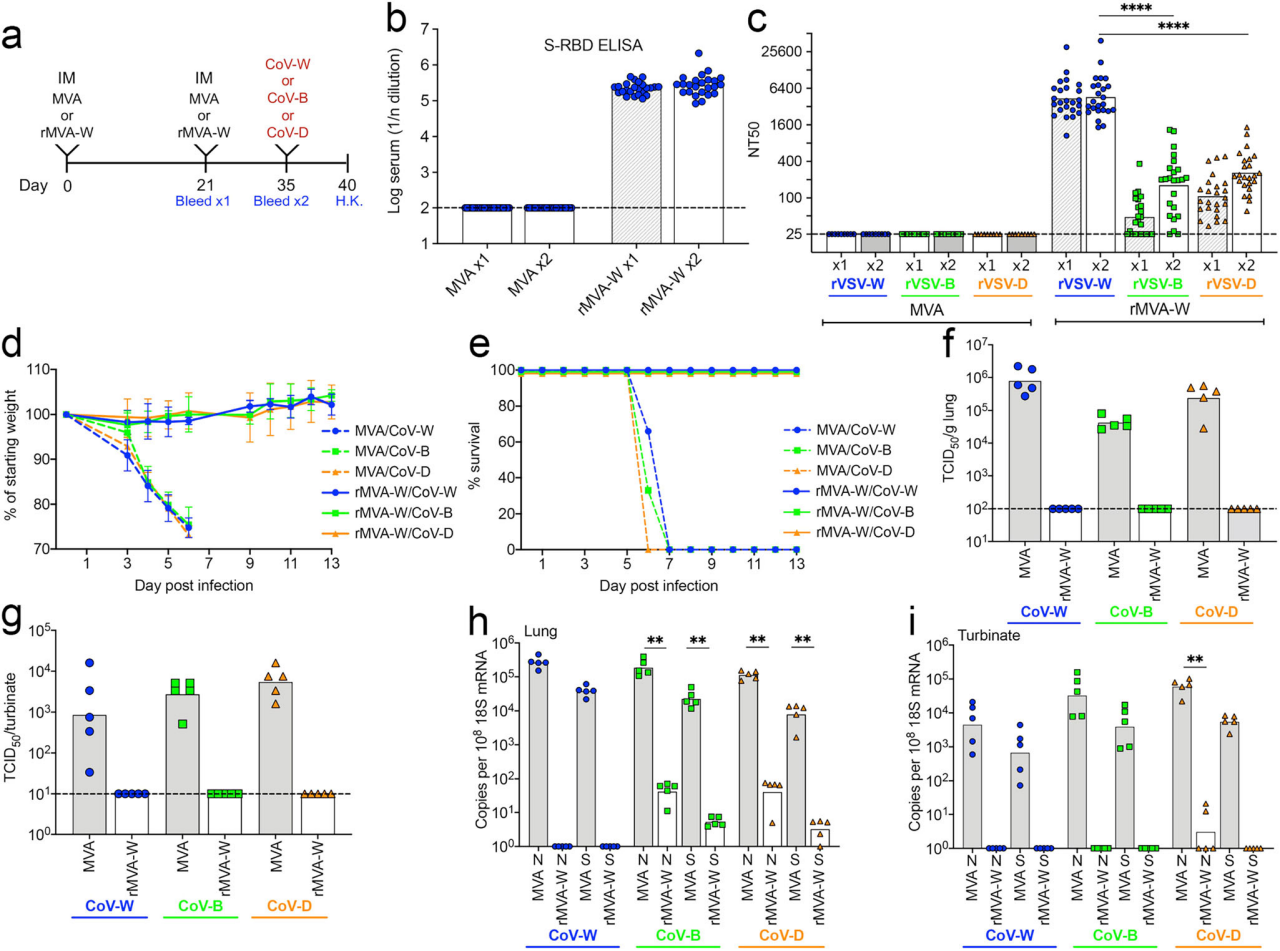
Protective immunity to SARS-CoV-2 variants following IM vaccination with rMVA-W. **a** Timeline of K18-hACE2 mice vaccinated IM on days 0 and 21 with 2 × 10^7^ PFU of MVA control (*n* = 24) or rMVA-W (*n* = 24). On day 35, the mice vaccinated with MVA and rMVA-W were each divided into 3 groups of *n* = 8 and each group was challenged with 10^4^ TCID_50_ of CoV-W, -B, or -D and 5 mice from each group were humanely killed 5 days later. The remaining 3 mice from each group were followed for weight loss and morbidity. **b** Serum antibody binding to the Wuhan S RBD and **c** neutralization of pseudoviruses rVSV-W, -B and -D at 3 and 5 weeks after vaccination were carried out with all MVA vaccinated mice (*n* = 24) and all rMVA-W vaccinated mice (*n* = 24). **d** Time course of weight loss and **e** survival on days after challenge with CoV-W, CoV-B or CoV-D were determined for 3 mice from each challenge group. Recovery of SARS-CoV-2 from **f** lungs and **g** nasal turbinates on day 5 after challenge of 5 mice from each challenge group. Copies of sgRNAs N and S relative to 18 S RNA from **h** lungs and **i** nasal turbinates on day 5 of the same mice analyzed in **f** and **g**. D day, HK humanely killed. Statistics: for **c** one way ANOVA with Tukey post-hoc test; for panels H, I Mann-Whitney. Error bars, SD; **p ≤ 0.002; ****p < 0.0001. Significance was not calculated when values of one group were all below the limit of detection. Dotted line, limit of detection.

Human vaccine studies indicate that the original SARS-CoV-2 vaccines provide incomplete protection against successive waves of variants. To begin to model this situation in the laboratory, K18-hACE2 mice were vaccinated IM twice 3 weeks apart with parental MVA as a control or with the same dose of rMVA-W and challenged with CoV variants 2 weeks later (Fig. 1a). Antibody binding to the Wuhan RBD was detected by ELISA after the first immunization and was increased only slightly by the second (Fig. 1b). The ability of the anti-Wuhan serum to neutralize variants was determined using rVSV-W, -B, and -D pseudoviruses, named for the strain of the spike protein that they express (Supplementary Fig. 1). The neutralization titers increased substantially after the second immunization for the vaccine mismatched rVSV-B and rVSV-D but not for the matched rVSV-W, which was already high (Fig. 1c). Neutralization of rVSV-W was higher than neutralization of rVSV-B and rVSV-D and the order of neutralization after both the prime and boost as determined by the Graphpad Prism Test for Linear Trend was rVSV-W > -D > -B.

Following challenge with CoV-W, -B or -D, all mice that received the control MVA vector lost weight and died or were euthanized because of 30% or more weight loss by day 6, whereas mice that received rMVA-W lost no weight and survived infection with each of the variants (Fig. 1d, e). To determine virus load, five additional mice in each group were humanely killed (H.K.) on day 5 for analysis of virus titers in internal organs. Substantial amounts of each of the SARS-CoV-2 viruses were detected by infectivity assays in the lungs and turbinates of control mice that received the MVA vector, whereas none was detected in mice that received rMVA-W regardless of the challenge strain of virus (Fig. 1f, g).

Analysis of sgRNAs provides an alternative and more sensitive assay for replication than the titer of infectious SARS-CoV-2. Of the sgRNAs, sgN is most abundant followed by sgS^22^. Digital droplet polymerase chain reaction (ddPCR) was used to detect sgN and sgS in our study and the values relative to 18S ribosomal RNA in each sample were calculated to correct for differences in organ weights and RNA recoveries. The forward and reverse primers were designed to preferentially copy sgRNAs rather than virion RNA because of discontinuous transcription so that gene expression is specifically detected by this assay^23^. High levels of sgN and sgS RNAs were detected in the lungs of the control mice that received the MVA vector following challenge with each of the variants. (Fig. 1h). In contrast, sgRNAs were not detected in mice immunized with rMVA-W and challenged with CoV-W and the levels were significantly diminished relative to the controls in mice challenged with CoV-B or -D (Fig. 1h). High titers of sgRNAs were also present in the nasal turbinates of challenged mice that had been immunized with the control MVA but were undetected or significantly diminished in mice that had been immunized with rMVA-W (Fig. 1i). These results indicated that a vaccine expressing the Wuhan S and administered IM protected against weight loss and death and significantly reduced the replication of CoV-B and -D by day 5, despite lower levels of neutralizing antibody to their S proteins. Nevertheless, virus replication in the lungs as determined by sgRNAs was lowest when the S proteins of the vaccine and challenge were matched. These results are consistent with the greater protection by current human vaccinees against severe disease than against mild infection by variant SARS-CoV-2 strains.

### Duration of cross-protective immunity following IM vaccination with rMVA-W

**Fig. 2:**
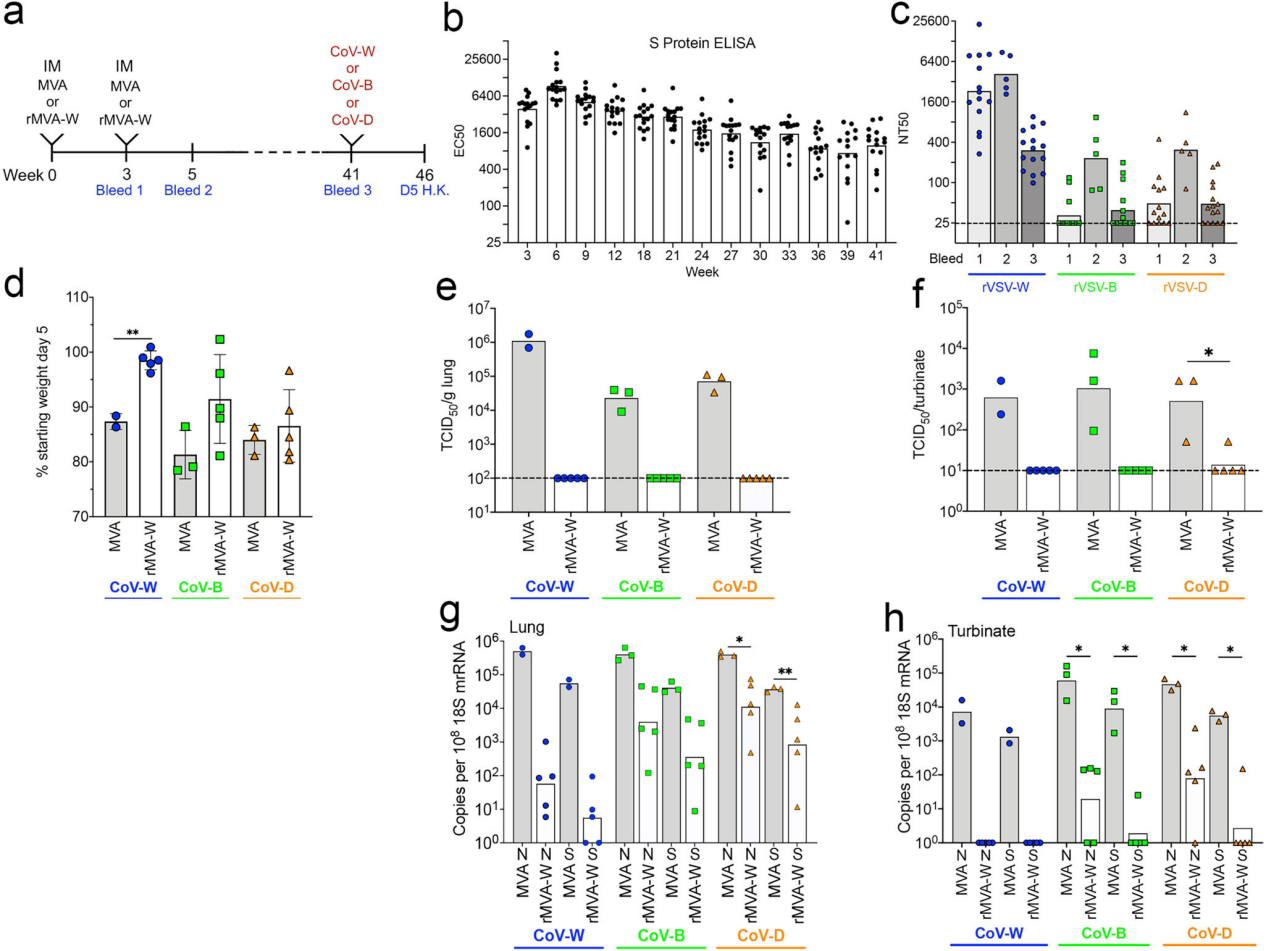
Duration of cross-protective immunity following IM vaccination with rMVA-W. **a** Timeline of K18-hACE2 mice vaccinated IM twice with 2 × 10^7^ PFU of MVA control (*n* = 8) divided into groups of 2–3 or rMVA-W (*n* = 15) divided into groups of 5 and challenged with 10^4^ TCID_50_ of CoV-W, -B, or -D approximately 9 months later. Mice were H.K. 5 days after challenge. **b** Binding of serum antibodies elicited by vaccination with rMVA-W to Wuhan S protein determined by ELISA (*n* = 15). **c** Neutralization of pseudoviruses rVSV-W, -B and -D by serum obtained at weeks 3, 5, and 41 (*n* = 15). **d** Weights of mice on day 5 after challenge relative to starting weights (MVA *n* = 2 or 3; rMVAs *N* = 5 per group). Recovery of SARS-CoV-2 from **e** lungs and **f** nasal turbinates on day 5 after challenge (n as in **d**). Copies of sgRNAs N and S relative to 18 S rRNA from **g** lungs and **h** nasal turbinates on day 5 after challenge (n as in **e**, **f**). Statistics: for D, F, H Welch’s t-test. *p ≤ 0.03; **p ≤ 0.002. Dotted line, limit of detection.

In the above experiment, mice were challenged at 2 weeks after vaccination when antibody levels woud be near their peak. To determine the duration of cross-protective immunity, additional groups of K18-hACE2 mice were vaccinated IM twice and held for about 9 months (Fig. 2a). Over this period, the binding to the Wuhan S protein decreased by about 70% (Fig. 2b). The neutralizing titer for each variant was boosted by the second immunization but then each also decreased substantially over time (Fig. 2c), consistent with the steep decline of neutralizing antibody in humans following mRNA and adenovirus vaccines^24^.

Challenges with CoV-W, -B and -D were carried out at week 41 (Fig. 2a). After challenge, mice that had received the control MVA vector lost ~10 to 20% of their starting weight by day 5; mice immunized with rMVA-W and challenged with CoV-W lost significantly less weight than controls; the mice challenged with CoV-B and -D also lost less weight than controls though there was considerable individual variation and the mean differences did not reach statistical significance (Fig. 2d). Mice that received the control MVA vector had substantial amounts of virus in the lungs and turbinates on day 5 following challenge, whereas mice that had been vaccinated with rMVA-W had little or no virus regardless of which SARS-CoV-2 strain was used for challenge (Fig. 2e, f). Considerable differences, however, were revealed by analysis of sgRNAs. Mice vaccinated with rMVA-W and challenged with CoV-W had >3-log mean reduction of sgRNAs in the lungs compared to controls whereas mice challenged with CoV-B and -D had 1- to 2-log mean reductions but with considerable individual variation that did not reach statistical significance in most cases (Fig. 2g). The control mice challenged with CoV-W also had considerable amounts of sgRNAs in the nasal turbinates, whereas none was detected in vaccinated mice challenged with CoV-W and the amounts were significantly reduced in mice challenged with CoV-B and -D (Fig. 2h). We concluded that protection was durable, but at a reduced level at 9 months and was greater when the rMVA-W vaccine and challenge virus were matched.

### Effect of IM immunizations with rMVA-W on inhibition of early stages of CoV variant infection

**Fig. 3:**
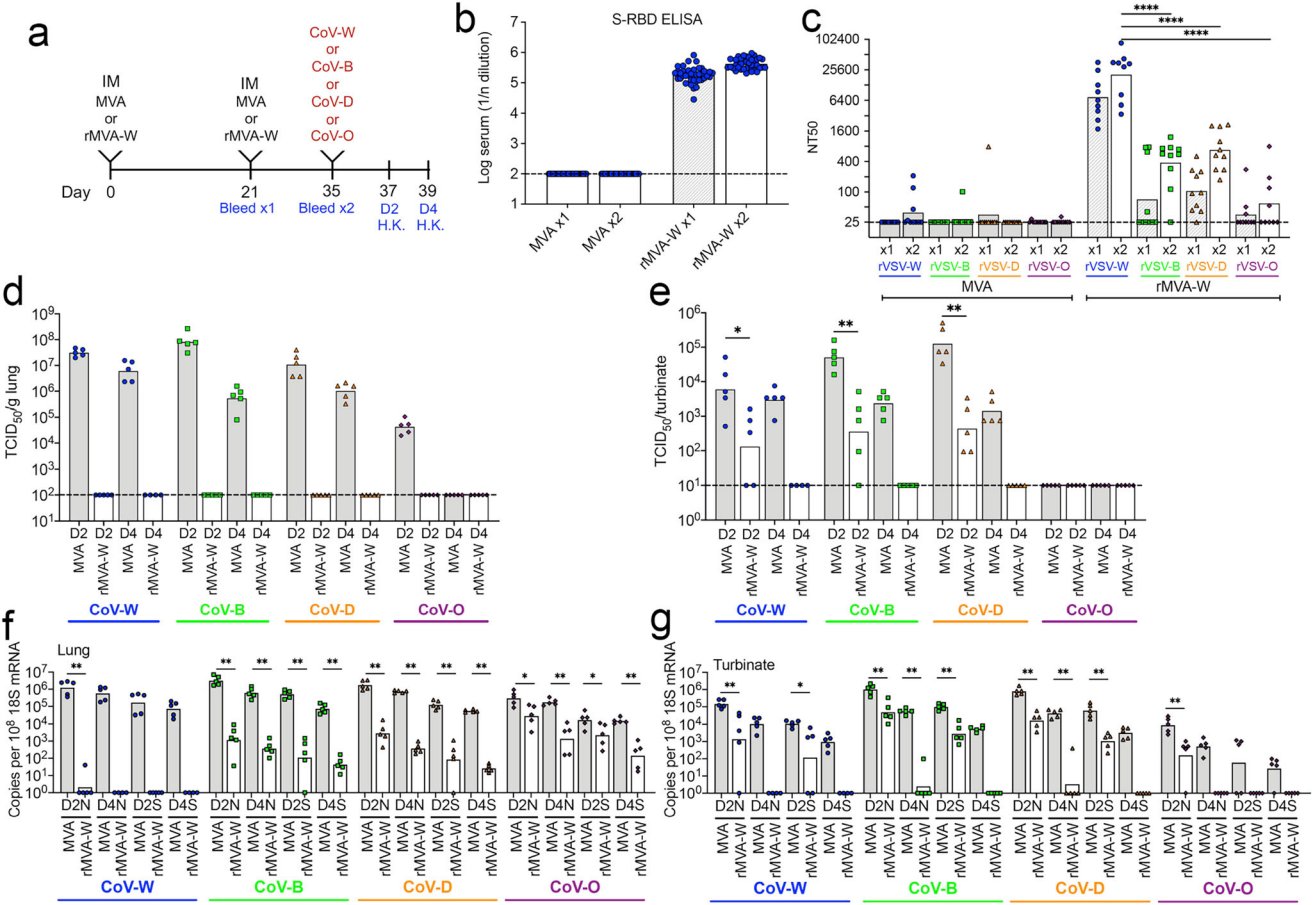
Inhibition of early stages of variant SARS-CoV-2 infections in mice immunized IM with rMVA-W. **a** Timeline of K18-hACE2 mice vaccinated IM twice with 2 × 10^7^ PFU of MVA control (*n* = 40) or rMVA-W (*n* = 40), divided into groups of 10 and challenged 2 weeks later with 10^4^ TCID_50_ of CoV-W, -B, -D or 5 × 10^4^ TCID_50_ of CoV-O. Half the mice were H.K. on day 2 and remainder on day 4. **b** Binding of serum antibodies to Wuhan RBD after first and second immunizations determined by ELISA (*n* = 40 per group). **c** Neutralization of pseudoviruses rVSV-W, -B, -D and -O by sera obtained after the first and second immunizations (*n* = 9 per group). Recovery of SARS-CoV-2 from **d** lungs and **e** nasal turbinates on days 2 (*n* = 5 per group) and 4 (*n* = 5 per group) after challenge. Copies of sgRNAs N and S relative to 18 S RNA from **f** lungs and **g** nasal turbinates on days 2 and 4 after challenge. (*n* = same as in **d** and **e**). Statistics: for **c** ANOVA with Tukey post-hoc; for **e**–**g** Mann-Whitney. *p ≤ 0.03; **p ≤ 0.002; ****p < 0.0001. Dotted line, limit of detection.

In the experiments described above, we determined virus loads and sgRNAs at 5 days after SARS-CoV-2 challenge when the infection had become well established in the controls. For the next experiments, we carried out analyses at 2 and 4 days after infection with SARS-CoV-2 to determine the progress of vaccination at earlier times (Fig. 3a). As found in our other experiments, binding of antibody to the Wuhan S RBD was detected after the first immunization and increased only slightly after the second (Fig. 3b). Neutralization titers for each of the pseudoviruses increased after the boost though less than half of the mice had antibodies that neutralized rVSV-O above the limit of detection (Fig. 3c). The titers were significantly higher for rVSV-W compared to rVSV-B, -D-, and -O and the order of neutralization after the prime and boost as determined by the Graphpad Prism Test for Linear Trend was rVSV-W > -D > -B > -O.

Following challenge of mice receiving the control MVA vector, the virus titers in the lungs were highest on day 2 and diminished to varying degrees on day 4 for each CoV variant (Fig. 3d). Notably, no virus was recovered from the lungs on either day from mice vaccinated with rMVA-W and challenged with CoV-W, -B, -D, or -O. Mice that received the control MVA vector and were subsequently challenged had substantial amounts of virus in the nasal turbinates on days 2 and 4 except for CoV-O, in which none was detected (Fig. 3e), consistent with the results obtained in our preliminary assessment of SARS-CoV-O in the K18-hACE2 model (Supplementary Fig. 2i). For mice vaccinated with rMVA-W, the CoV-W, -B or -D titers in the turbinates were significantly reduced relative to the controls on day 2 and undetectable on day 4. CoV was also undetectable in the turbinates of vaccinated mice in the previous experiment when day 5 was examined (Fig. 2f).

In contrast to the substantial decline in recovery of CoV from the lungs of mice between days 2 and 4, the amounts of sgRNAs declined only slightly (Fig. 3f). In addition, sgRNAs were detected after CoV-O infection whereas infectious virus was not. We had previously suggested that the difference between detection of infectious virus and sgRNAs might be due to neutralizing antibody in the tissue extracts inhibiting the former^18^. However, that is not the whole explanation because for CoV-O there was no detectable neutralizing antibody. Notably, mice that had been immunized with rMVA-W and challenged with CoV-W had little or no sgRNA in the lungs on either day and the levels were significantly reduced on both days after challenge with the other variants (Fig. 3f). In the nasal turbinates, there was a substantial decline of sgRNAs between days 2 and 4 of mice that received the control MVA vector and challenged with each of the variants including CoV-O. On day 2, sgRNAs were significantly reduced relative to controls in vaccinated mice challenged with CoV-W, -B, -D, or O and were undetected or barely detected on day 4 (Fig. 3g). These data indicated that IM vaccination with rMVA-W reduced replication of each of the variants but did not prevent infection as judged by the detection of virus and sgRNAs in the turbinates on day 2. Interestingly, although rMVA-W induced low Omicron neutralizing activity as measured in vitro, the Omicron sgRNAs were significantly reduced in rMVA-W vaccinated mice.

### Superior cross-protection achieved by IN immunizations with rMVA-W

**Fig. 4:**
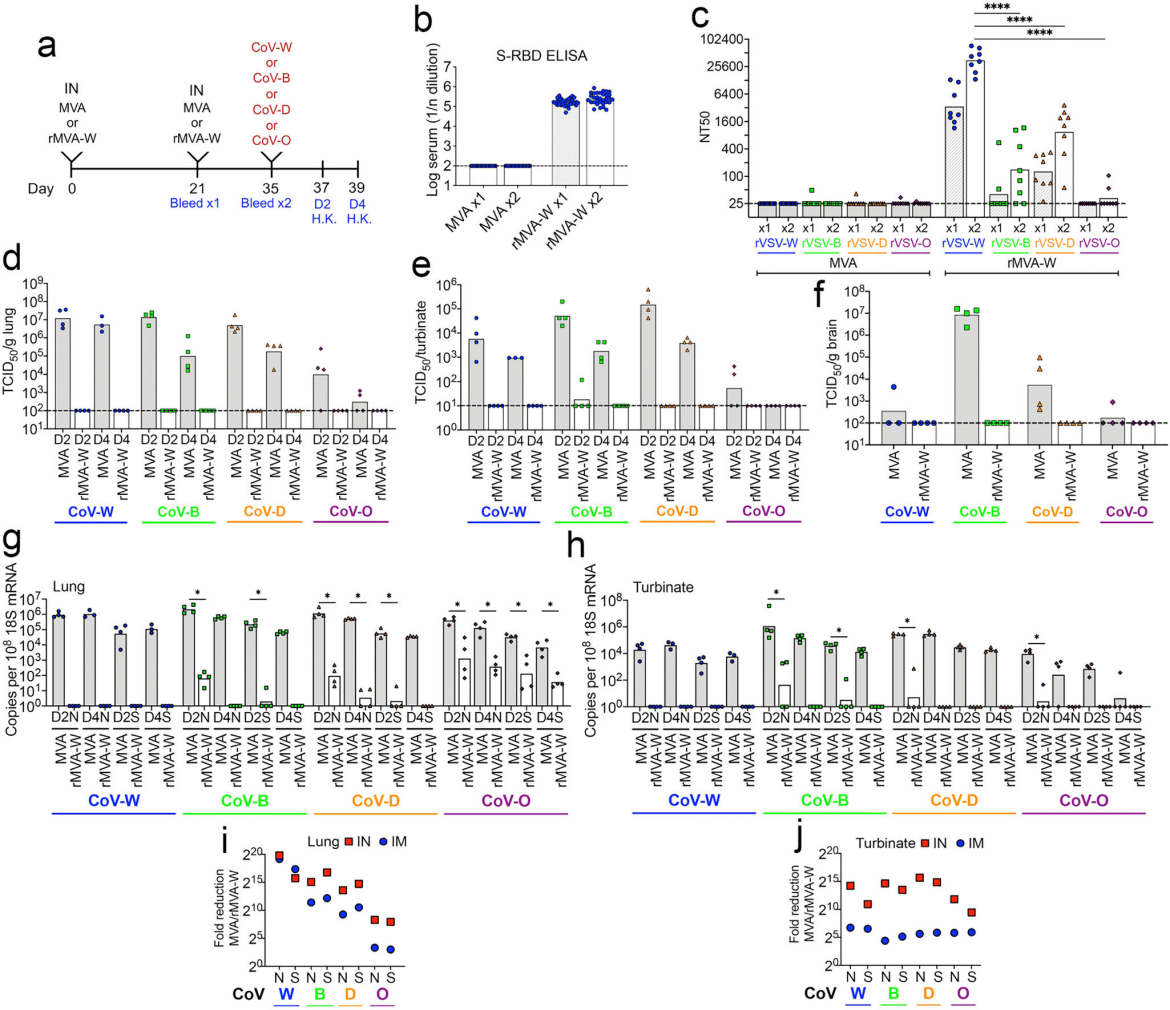
Inhibition of variant SARS-CoV-2 infections in mice immunized IN with rMVA-W. **a** Timeline of K18-hACE2 mice vaccinated IN twice with 2 × 10^7^ PFU of MVA control (*n* = 31) or rMVA-W (*n* = 32), divided into groups of 7 or 8 and 2 weeks later challenged with 10^4^ TCID_50_ of CoV-W, -B, or -D or 5 × 10^4^ TCID_50_ of CoV-O. Half the mice were H.K. on day 2 and remainder on day 4. **b** Serum antibody binding to Wuhan RBD determined by ELISA after the first and second immunizations. (*n*= MVA 31, rMVA 32). **c** Neutralization of pseudoviruses rVSV-W, -B, -D and -O by serum obtained after the first and second immunizations. (*n* = 8 per group). Recovery of SARS-CoV-2 from **d** lungs and **e** nasal turbinates on days 2 and 4 and **f** brain on day 4 after challenge. *n* = 3 or 4 per group. Copies of sgRNAs N and S relative to 18 S RNA from **g** lungs and **h** nasal turbinates on days 2 and 4 after challenge. Fold-reduction of sgRNAs in nasal turbinates on day 2 for mice immunized **i** IN and **j** IM. Data replotted from Figs. 3g and 4h. Statistics: for **c** ANOVA with Tukey post-hoc; for **g**, **h** Mann-Whitney. *p ≤ 0.03; **p ≤ 0.002; ****p < 0.0001. Dotted line, limit of detection.

There is interest in IN vaccination as a way of preventing or reducing the viral load in the upper respiratory tract. We previously reported that IN administration of rMVA-W prevented or more rapidly eliminated upper respiratory infection with CoV-W than when administered IM^18^. Here, we wanted to determine whether IN delivery is advantageous for cross-protection of variants, particularly when cross-neutralization is low. Mice were inoculated with the MVA control or rMVA-W as in previous sections, except that the route was IN (Fig. 4a). Antibody binding to the RBD of the Wuhan spike protein was detected after the first immunization with rMVA-W and increased slightly after the second (Fig. 4b) as we found for IM vaccination. However, boosting with rMVA-W increased neutralizing antibodies to rVSV-W, -B, and D but not to rVSV-O and the titers were significantly higher to rVSV-W compared to the others (Fig. 4c). The order of neutralization determined by the Graphpad Prism Test for Linear Trend was rVSV-W > D > B > O), as determined with IM vaccinations. Following challenge of mice that received the control MVA, the CoV-W and variant strains were detected in the lungs and turbinates on day 2 and decreased to varying extents on day 4 (Fig. 4d, e). A previous study had shown CoV-W in the brain of K18-hACE-2 mice on day 7 but not on day 3^25^. We examined the brains only on day 4 and found little CoV-W in the brains of the control MVA vaccinated mice, but unexpectidly found more CoV-B and CoV-D (Fig. 4f), suggesting more rapid spread of these variants. However, in mice vaccinated IN with rMVA-W, virtually no virus of any strain was detected on days 2 or 4 in lungs, nasal turbinates or brains of mice (Fig. 4d–f), whereas considerable virus had been detected on day 2 in the turbinates of mice that had been vaccinated IM (Fig. 3e). Because the blood-brain barrier normally prevents the passage of antibodies, the vaccines likely prevented virus spread before reaching the brain. sgRNAs were also undetectable on day 2 of the rMVA-W vaccinated mice challenged with CoV-W and were significantly reduced in the lungs of mice challenged with COV-B, -D and -O (Fig. 4g). On day 4 there was little or no sgRNAs in the lungs of IN-vaccinated mice following challenge with CoV-W, -B or -D and the amounts were significantly reduced after challenge with CoV-O compared to controls (Fig. 4g). Moreover, sgRNAs were undetectable or detected at very low levels in the turbinates of mice infected with any of the variants on day 2 and none was detected on day 4 (Fig. 4h), in contrast to the results obtained by IM vaccination (Fig. 3g). Direct comparison of the data for day 2 in Fig. 3f, g with the data in Fig. 4g, h revealed that mice immunized IN rather than IM had a greater reduction in sgRNAs of each variant that was more pronounced in the turbinates (Fig. 4j) than the lungs (Fig. 4i). Thus, IN vaccination with rMVA-W was advantageous for protection of the upper respiratory tract against variant as well as matched challenges.

### Construction and immunogenicity of rMVAs with variant spikes

**Fig. 5:**
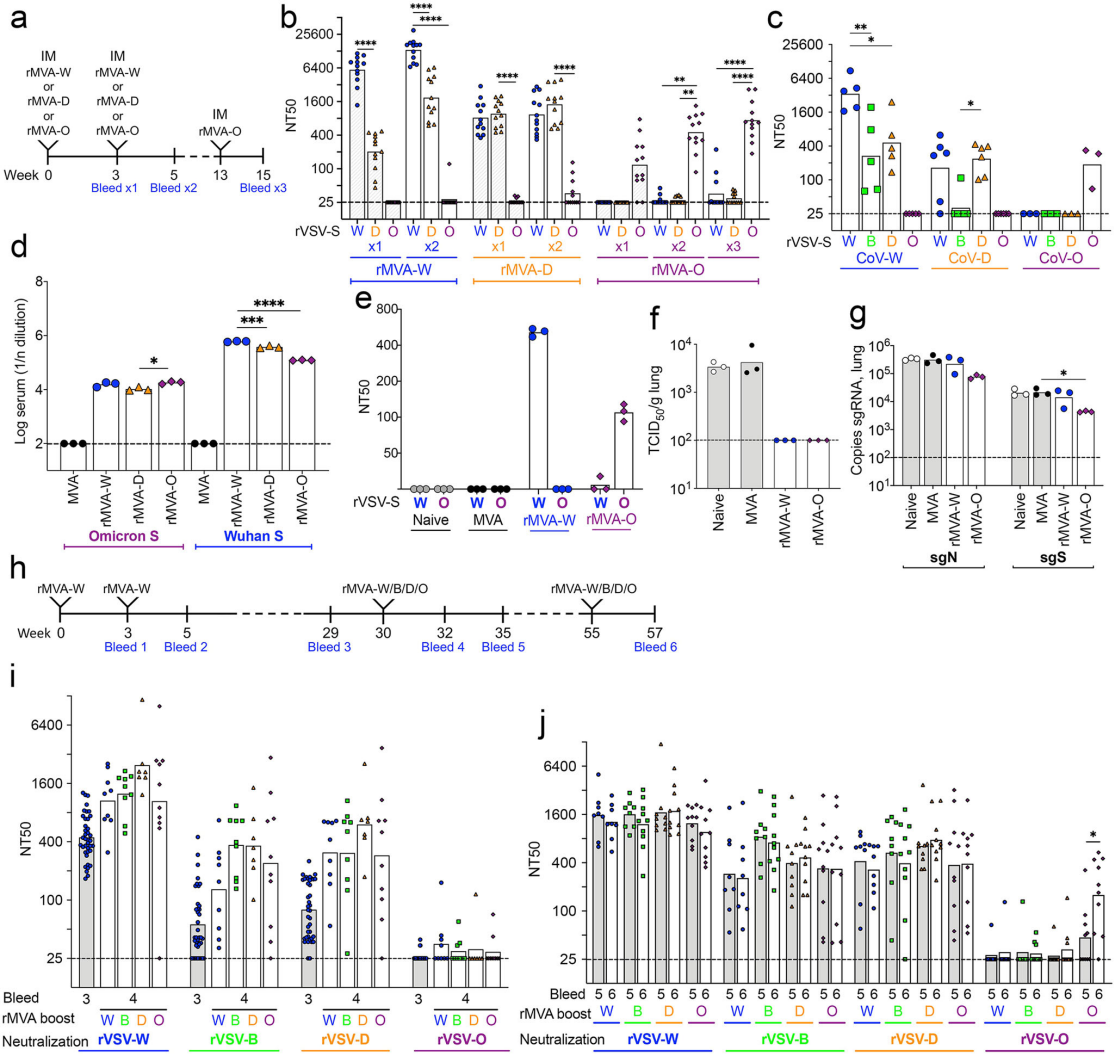
Neutralizing antibody responses following boosts with rMVAs expressing matched or mismatched S. **a** Timeline of matched prime and boost IM vaccinations of C57BL/6 mice with 2 × 10^7^ PFU of MVA or rMVA-W, -D, and -O. (*n* = 10 per group). **b** Neutralization of pseudoviruses rVSV-W, -D and -O by serum obtained after the prime with rMVA-W, -D and -O and after the matched boost. *n* = 10 per group. **c** Neutralization of pseudoviruses by sera from mice sublethally infected with CoV-W, CoV-D or CoV-O in Supplementary Fig. 2. *n* = 3 to 5. **d** Binding of pooled serum from bleed 2 to Wuhan and Omicron S determined by ELISA. *n* = 3 per group. **e** Serum neutralizing titers of mice one day after receiving pooled serum IP from naive mice or mice immunized twice with MVA, rMVA-W or rMVA-O. *n* = 3. Recovery of **f** SARS-CoV-2 and **g** sgRNAs from the lungs at 4 days after challenge of passively immunized mice. **h** Timeline of C57BL/6 mice were immunized twice with rMVA-W and boosted with rMVA-W, -B, -D, or -O. *n* = 9 for rMVA-W and *n* = 10 for other rMVAs. **i** Neutralization of pseudoviruses rVSV-W, -B, -D and -O by serum obtained after the third immunization (bleed 4). *n* = 9 for rMVA-W and *n* = 10 for other rMVAs. **j** Neutralization of pseudoviruses by sera obtained after the fourth immunization (bleed 6). *n* = 9 for rMVA-W and *n* = 10 for other rMVAs. Statistics: for **b**–**d** ANOVA with Dunnetts post-hoc. *p ≤ 0.03; **p ≤ 0.002; ***p ≤ 0.0002; ****p < 0.0001. Dotted line, limit of detection.

Thus far, we immunized mice with rMVA expressing the Wuhan S protein and challenged with variant CoV-2, representing the situation that followed the original SARS-CoV-2 vaccines. For the next experiments, we constructed rMVAs expressing the S proteins of variant strains to see if they provided better protection. S protein expression was verified by analysis of infected HeLa cells by Western blotting using antibody to the FLAG tag, which is fused to S, green fluorescent protein that is encoded as a separate protein in the rMVA genome and anti-Wuhan RBD (Supplementary Fig. 3a–c). Some variation in RBD binding could be due to S sequence differences. Cell surface expression of the variant S proteins and binding to hACE2 were demonstrated by flow cytometry (Supplementary Fig. 3d). To compare their immunogenicity, C57BL/6 mice were vaccinated IM with rMVAs expressing variant S proteins followed by a boost with the same rMVA used for the initial vaccination and the neutralization of pseudoviruses expressing spike proteins matched or mismatched to the vaccines were determined (Fig. 5a, b). Sera from mice immunized with rMVA-W neutralized rVSV-W better than rVSV-D and had no detectable activity against rVSV-O. After the second vaccination with rMVA-W, neutralizing antibody to rVSV-W and -D were increased but most of the mice still made no detectable neutralizing antibody to rVSV-O. Sera from mice vaccinated with rMVA-D neutralized rVSV-W almost as well as rVSV-D but also had low neutralizing activity to rVSV-O and only small increases occurred after the second immunization. Sera from mice that received rMVA-O had little or no neutralizing activity to rVSV-W or -D but had detectable activity against the matching rVSV-O. After a second and third rMVA-O vaccination, neutralization of rVSV-O was boosted but the neutralizing titers to rVSV-W and -D were minimally increased (Fig. 5b).

The S proteins expressed by the rMVA vectors had been modified to stabilize the pre-fusion form, prevent furin cleavage and increase cell surface expression as described previously^12^. To be certain that these changes were not responsible for diminished cross-neutralizing activity, we obtained sera from the K18-hACE2 mice that had been sublethally infected with CoV-W, CoV-B, CoV-D or CoV-O in the experiment depicted in Supplementary Fig. 2. The NT50 values were lower than that achieved by rMVA vaccination but the relative cross-neutralizing activities of these serum samples were similar e.g., sera from mice infected with each CoV variant had the highest neutralizing activity against the rVSV with the corresponding S (Fig. 5c).

The low cross-neutralizing activity raised the question of the basis for cross-protection, particularly for Omicron. One consideration is that in contrast to the low ability of sera from mice immunized with rMVA-W to neutralize rVSV-O, the rMVA-W sera bound as well to Omicron S as rMVA-O sera, although rMVA-D sera bound slightly less (Fig. 5d). Sera from mice immunized with rMVA-O and -D also exhibited substantial binding to Wuhan S, although significantly less than sera from mice immunized with rMVA-W (Fig. 5d). We considered that the relatively greater binding of sera to mismatched S proteins compared to their neutralizing ability may have significance for cross-protection in vivo. To investigate this, a passive transfer experiment was carried out by intraperitoneal inoculation of pooled sera from mice vaccinated IM with rMVA-W or rMVA-O into K18-hACE2 mice. One day after serum transfer and just before challenge with CoV-O, the NT50 values of the sera from rMVA-W-vaccinated mice were >400 for rVSV-W and undetectable for rVSV-O (Fig. 5e). The corresponding NT50 values for the mice receiving pooled sera from rMVA-O-vaccinated mice were >100 for rVSV-O and undetectable or barely detectable for rVSV-W (Fig. 5e). Because of limitations of passive transfer, we were not able to administer sufficient serum to raise the NT50s of matched sera to the levels achieved with active vaccination. Despite the difference in neutralizing titers, no CoV-O was recovered from the lungs at 4 days after challenge of mice receiving anti-Wuhan or anti-Omicron sera (Fig. 5f). However, the mice that received anti-Omicron serum also had slightly reduced sgN and sgS RNAs in the lungs, whereas the mice that received anti-Wuhan serum did not (Fig. 5g). These data indicated that the anti-Wuhan serum without detectable in vitro neutralizing activity for Omicron was partially protective in vivo as judged by the reduction of virus in the lungs.

### Neutralizing antibody responses to mismatched prime and boost vaccinations

Another question is whether heterologous boosting with variant spike proteins will increase neutralizing activity to the original spike protein, the variant or both. To investigate this, C57BL/6 mice were vaccinated twice with rMVA-W. After 30 weeks, the mice were bled and groups of 9 to 10 mice were re-vaccinated with rMVA-W, -B, -D or -O (Fig. 5h). The neutralizing titer against the original immunogen rVSV-W as well as rVSV-B and rVSV-D increased after boosting with each of the variant rMVAs with the exception of rVSV-O (Fig. 5i). Because of the low neutralization titer against Omicron obtained following immunization twice with rMVA-W and once with rMVA-O or other rMVA variants, we decided to boost all the mice again with the same rMVAs used in the previous boost. None of the boosts increased the neutralizing titer to rVSV-W, rVSV-B or rVSV-D. However, the rMVA-O boost significantly increased the neutralizing titer to rVSV-O, whereas none of the other boosts did (Fig. 5j). These results indicated that a second booster immunization with rMVA-O is beneficial both for naive mice as well as mice primed with the rMVA expressing the ancestor S protein.

### Protective immunogenicity of rMVAs expressing variant S proteins

**Fig. 6:**
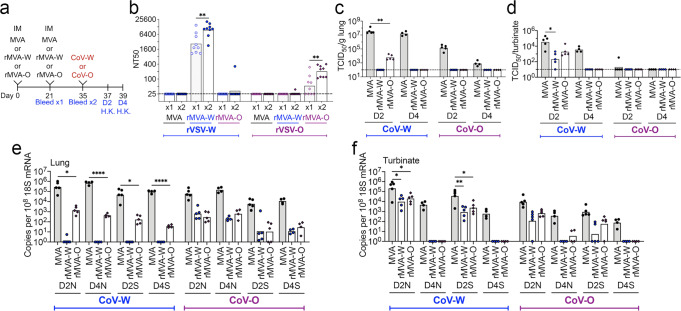
Protection of mice immunized IM with rMVAs to matched or mismatched CoVs. **a** Timeline showing IM immunizations of K18-hACE2 mice (*n* = 9 per group) with 2×10^7^ PFU of MVA, rMVA-W or rMVA-O and matched and mismatched challenges with 10^4^ TCID50 of CoV-W and 5x10^4^ TCID_50_ of CoV-O. Mice were H.K on day 2 (*n* = 5) and day 4 (*n* = 4). **b** Neutralization of pseudoviruses rVSV-W and -O by serum obtained after one and two IM immunizations with MVA, rMVA-W or rMVA-O. n = 9 per group. Recovery of SARS-CoV-2 from **(c)** lungs and **(d)** nasal turbinates on days 2 and 4 after challenge with CoV-W or CoV-O. Copies of sgRNAs N and S relative to 18 S RNA from **(e)** lungs and **(f)** nasal turbinates on days 2 and 4 after challenge. Statistics: for panels b and c Mann-Whitney; d-f ANOVA with Dunnet post-hoc. *p ≤ 0.03; **p ≤ 0.002; ****p < 0.0001. Dotted line, limit of detection.

In further studies, we focused on CoV-W and CoV-O because they represent variants that arose early and late during the pandemic with numerous differences in their S proteins. The vaccination and challenge schedule is outlined in Fig. 6a. As also shown earlier in this study, immunization of K18-hACE2 mice with rMVA-W elicited high neutralizing antibody to rVSV-W and little to rVSV-O, whereas immunization with rMVA-O only elicited neutralizing antibody to rVSV-O (Fig. 6b). Nevertheless, immunization with rMVA-W reduced the titers of both CoV-W and CoV-O in the lungs compared to the controls on day 2 and both viruses were undetectable on day 4 (Fig. 6c). Immunization with rMVA-W also reduced the titer of CoV-W in the nasal turbinates but the level of CoV-O in the turbinates was too low to determine vaccine efficacy (Fig. 6d). Likewise, immunization with rMVA-O reduced both CoV-W and CoV-O in the lungs (Fig. 6c) and CoV-W in the turbinates (Fig. 6d).

Analysis of sgRNAs provided a more discriminative comparison of the protection afforded by the different immunizations. Immunization with rMVA-W provided complete protection of the lungs from CoV-W on days 2 and 4, whereas immunization with rMVA-O provided significant but partial protection (Fig. 6e). However, the two vaccines provided similar 2-log reduction of sgRNAs in the lungs of mice infected with CoV-O (Fig. 6e). In the nasal turbinates, sgRNAs were reduced on day 2 and undetectable on day 4 after challenge with CoV-W regardless of whether the mice were immunized with rMVA-W or rMVA-O (Fig. 6f). CoV-O sgRNAs were also reduced by similar amounts in the nasal turbinates when vaccinated with either rMVA-W and rMVA-O. These data indicated that considerable protection can occur even if low neutralizing antibody is induced.

**Fig. 7:**
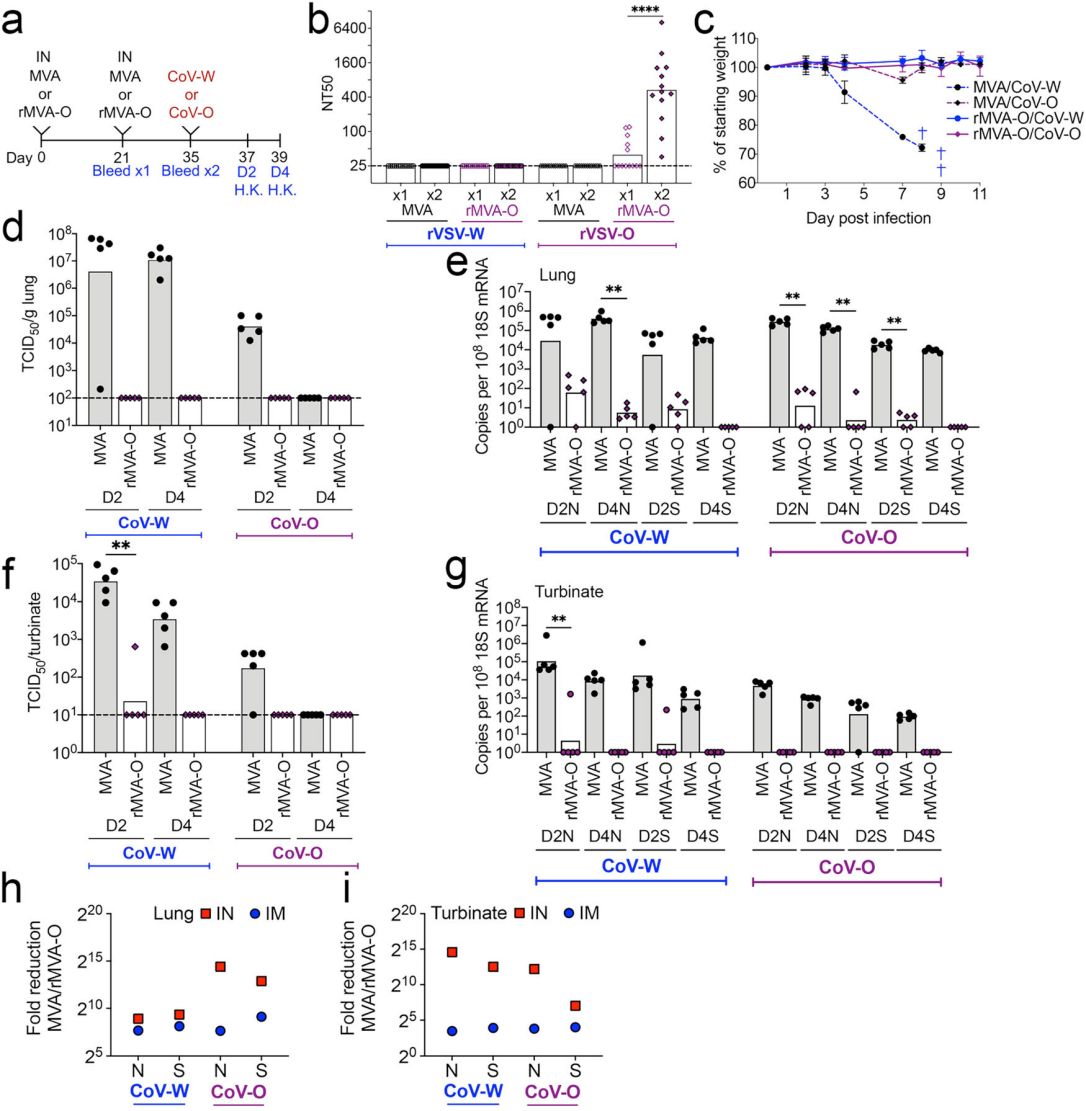
Protection of CoV-W and CoV-O challenged mice vaccinated IN with rMVA-O. **a** Timeline of IN immunizations of K18-hACE2 mice (*n* = 13 per group) with 2 × 10^7^ PFU of MVA or rMVA-O and matched and mismatched challenges with 10^4^ TCID_50_ of CoV-W and 5 × 10^4^ TCID_50_ of CoV-O. On days 2 and 4, 5 mice of each group were H.K. and 3 were followed for weight loss. **b** Neutralization of pseudoviruses rVSV-W and -O by serum obtained after one and two IN immunizations with MVA or rMVA-O. *n* = 13 per group. **c** Weights of mice following challenge with CoV-O or CoV-W. *n* = 3 per group. Recovery of **d** virus and **e** sgRNAs in the lungs of mice challenged with CoV-W or CoV-O. *n* = 5 per group. Recovery of **f** virus and **g** sgRNAs in the nasal turbinates of mice challenged with CoV-W or CoV-O. *n* = 5 per group. Fold-reduction of sgRNAs in lungs **h** and **i** nasal turbinates on day 2 for mice immunized IN and IM. Statistics: **b**, **d**–**g** Mann-Whitney. Error bars, S.D.; *p ≤ 0.03; **p ≤ 0.002; ***p ≤ 0.0002; ****p < 0.0001. Dotted line, limit of detection.

Finally, we investigated the use of rMVA-O as a nasal vaccine for protection against CoV-O and CoV-W (Fig. 7a). Low neutralizing activity to rVSV-O was detected in serum from about half the mice immunized once with rMVA-O that increased to all mice immunized twice (Fig. 7b). However, neutralization of rVSV-W was undetectable after the first or second vacciniation with rMVA-O similar to IM vaccination. The control mice challenged with CoV-W succumbed to the infection, whereas mice vaccinated IN with rMVA-O had no weight loss following challenge with CoV-W (Fig. 7c). Because of the low pathogenicity of CoV-O, weight loss could not be used to discern the extent of homologous protection (Fig. 7c). However, both CoV-O and CoV-W were detected in the lungs of the control mice, whereas little to none of either was found in the mice that were vaccinated with rMVA-O (Fig. 7d). Moreover, sgRNAs were significantly diminished in the lungs of mice challenged with either CoV-W or CoV-O (Fig. 7e). Most striking was the almost complete reduction of virus (Fig. 7f) and sgRNAs (Fig. 7g) in the nasal turbinates of mice immunized with rMVA-O and challenged with CoV-W or CoV-O. The relative reduction of sgRNAs achieved by IM and IN vaccination with CoV-O was determined by comparing the data on day 2 from Fig. 6e, f and Fig. 7e, g. IN vaccination with CoV-O reduced the sgRNAs in the lungs following CoV-O infection but the route of vaccination has less of an effect on the sgRNAs following CoV-W challenge (Fig. 7h). However, the greater effect of IN administration was more evident by analysis of sgRNAs in the nasal turbinates following both CoV-W and CoV-O challenge (Fig. 7i). These data highlight the efficacy of IN administration for protection of the upper respiratory tract by vaccines matched and mismatched to the challenge CoV.

## Discussion

The SARS-CoV-2 pandemic has entered a phase in which large segments of the population have some immunity due to previous infection or vaccination. While there has been a drop in serious disease and hospitalization, variants continue to arise and spread. Using an inbred mouse model expressing hACE2, we investigated several topics related to improving vaccine efficacy in the current situation, including cross-neutralization of variants, boosting with variant S proteins, role of non-neutralizing antibody and particularly enhanced protection by IN administration of matched and mismatched vaccines. We used MVA, an attenuated vaccinia virus vector, that has been extensively used for immunological studies and is currently in clinical vaccine trials for a variety of infectious diseases including SARS-CoV-2. Before carrying out these investigations, we compared the abilities of variant SARS-CoV-2 strains to infect susceptible K18-hACE2 mice. Whereas, Washington, Beta and Delta strains were highly lethal, Omicron was less so and lower titers of the latter virus were recovered from the upper and lower respiratory tract. Although the basis for the lower virulence of CoV-O is unexplained, it is in accord with other studies in mice and hamsters^20,21^. However, we found the amounts of sgRNAs in the lungs and nasal turbinates of the different strains to be more similar allowing a better comparison of their replication. In order to carry out our study, we constructed a panel of rMVA vaccines and a panel of rVSV pseudoviruses expressing the variant S proteins.

Initially we focused on the ability of rMVA-W, expressing the ancestor Wuhan S protein, to induce cross-neutralizing antibodies. Repeated immunizations with rMVA-W increased neutralization titers to Delta and Beta but hardly to Omicron, which has the most divergent S-protein. We found that neutralization was in the order of Wuhan > Delta > Beta > Omicron. The same order was found in macaques that had been vaccinated with mRNA expressing the Wuhan S^26^. The same order was also found when K18-hACE2 mice were infected with CoV-2 variants in this study, corroborating an earlier study^21^. Moreover, sera from humans vaccinated with mRNA expressing the Wuhan S has a greatly diminished ability to neutralize Omicron^27^. Nevertheless, vaccination with rMVA-W protected hACE2 mice against weight loss and death and reduced virus replication in the upper and lower respiratory tracts. However, whereas no replication of the ancestral strain of SARS-CoV-2 was detected in the lungs by sensitive sgRNA analysis, some replication of other strains was found though significantly reduced compared to controls. Although rMVA-W elicited little anti-Omicron neutralizing antibody, there was appreciable Omicron S-binding antibody that provided partial protection when passively transferred to mice. In another study, Kaplonek and co-workers^28^ determined that an mRNA SARS-CoV-2 vaccine expressing ancestral S induced antibodies that maintain Fc effector functions across variants, which could explain the protection seen here. In addition, several studies have shown that vaccines elicit highly conserved cellular immunity to CoV-O^29–31^. We had previously reported that rMVA-W stimulated antigen-specific T cells^12^ in K18-hACE2 mice and the majority of the peptides in the positive pools are present in the variant S proteins. The conclusion from this phase of the study was that the mouse model mirrored clinical experience in that immunity to the ancestral SARS-CoV-2 protected against severe disease by variants but only partially prevented infection and replication.

We considered several approaches to providing more complete protections against variant CoV strains. The first was to construct vaccines expressing variant S proteins. When we immunized mice with rMVAs-W, -D or -O, neutralization of the matched pseudovirus was greater than mismatched. The difference was least between Wuhan and Delta and greatest between Omicron and all others. Indeed, even repeated repeated immunizations with rMVA-W induced minimal neutralization of rVSV-O and repeated immunization with rMVA-O elicited minimal neutralization of the other variants. Low or absent cross-neutralizing antibodies were also reported following immunization with mRNA encoding Omicron S^32,33^. Furthermore, even though rMVA-O induced antibodies that significantly neutralized rVSV-O, the titers were less than those elicited by rMVA-W for Wuhan or rMVA-D for Delta. Diminished cross-neutralizing antibodies were also found in sera from hACE2 mice infected with sublethal doses of SARS-CoV-2 variants demonstrating that this was not a problem with the rMVA or mRNA vaccines. Suryawanshi et al.^21^ also reported that sera from mice infected with CoV-O only neutralized CoV-O and not other variants. These data support the idea of a need for continual development of new vaccines based on evolving S proteins^8^.

Since a high proportion of the population have immunity to older strains of CoV due to vaccination or infection, a pertinent question was whether boosting mice that had been vaccinated with rMVA-W with rMVA-B, -D, or -O would increase antibodies to Wuhan S (original antigenic sin), to the variants or both. Following two vaccinations with rMVA-W, a single vaccination with rMVA-B or rMVA-D boosted the neutralization titers to Wuhan as well as to self. Although a single immunization with rMVA-O boosted neutralizing antibody to the other variants, a second vaccination with rMVA-O was required to induce neutralizing antibody to itself. Thus, two rMVA-O vaccinations were needed to raise Omicron neutralizing antibody in both naive mice and mice that had been previously vaccinated with rMVA-W. Omicron mRNA vaccines were also reported to boost neutralizing antibodies to the original vaccine as well as to Omicron^32,33^. Although not directly measured in our study, it seems likely that the first Omicron vaccination elicited Omicron-specific memory cells that were activated on the second vaccination. Boosting humans with a bivalent Wuhan and Omicron BA.1-containing mRNA vaccine induced neutralizing antibodies to both strains^8^. Based on our mouse studies, we would predict a further increase in omicron-neutralizing antibody after another boost with the bivalent vaccine.

By analyzing virus and sgRNAs in nasal turbinates and lungs at 2 and 4 days after SARS-CoV-2 infection of K18-hACE2 mice, we confirmed our previous data on the better protection afforded by IN compared to IM vaccination with rMVA-W. In the latter study, induction of antigen-specific IgA and higher numbers of CD8 + T cells were found in the lungs. Here we showed that IN vaccination also provided greater protection against other SARS-CoV-2 variants following immunization with matched as well as mismatched S vaccines. Although our studies point out the feasabilty of an rMVA-based intranasal vaccine, other attenuated viruses are also being considered. Indeed, a search of Clinicaltrials.gov revealed proposed trials of Newcastle disease virus, parainfluenza virus 5, respiratory syncytial virus, influenza virus, adenovirus as well as MVA vectored SARS-CoV-2 spike protein intranasal vaccines.

## Methods

### Mice

Five- to six-week-old female C57BL/6ANTac and B6.Cg-Tg(K18-hACE2)2Prlmn/J mice were obtained from Taconic Biosciences and Jackson Laboratories, respectively. Typically, 3–5 mice were housed per sterile, ventilated microisolator cage in an ABSL-2 or ABSL-3 facility. Female mice were used because males would have had to be caged separately to prevent aggressive behavior.

### Cells

Vero E6 cells (ATCC CRL-1586) and Vero E6 hTMPRSS2 hACE2^18^ were maintained in Dulbecco’s Modified Eagle Medium supplemented with 8% heat-inactivated fetal bovine serum, 2 mM L-glutamine, 10 U/ml penicillin, and 10 µg/ml streptomycin.

### rMVAs

DNA encoding the SARS-CoV-2 strain Beta, Delta and Omicron S proteins modified as in Supplementary Fig. 1 were inserted into pLW44 insertion vector using the *Xma*I and *Sal*I sites under the control of the VACV modified H5 early late promoter and adjacent to the gene encoding enhanced GFP regulated by the VACV P11 late promoter^12^. All rMVA viruses were purified by two consecutive sedimentations through sucrose cushions. Vaccinations were carried out with the same preparations of MVAs and rMVAs making it possible to directly compare experiments in different figures.

### Vaccination

A total of 2 × 10^7^ PFU of rMVA in phosphate buffered saline and 0.05% bovine serum albumin was injected in two 50 µl aliquots IM into each hind leg of the mouse. For IN vaccination, mice were lightly sedated with isoflurane and 2 × 10^7^ PFU of rMVA in 50 µl was administered into one nostril.

### Infection with SARS-CoV-2

SARS-CoV-2 USA-WA1/2020 from BEI resources (Ref# NR-52281) was propagated in Vero cells (CCL81); SARS-CoV-2 Alpha (UK/CA B.1.1.7) and SARS-CoV-2 Beta (RSA 1.351 501Y) from the NIAID Integrated Research Facility at Ft. Detrick; SARS-CoV-2 Delta (hCoV-19/USA/MD – HP05285/2021 VOC G/478 K.V1 B.1.617.2+AY.1+AY.2) from Andrew Pekosz at Johns Hopkins University, and SARS-CoV-2 Omicron BA.1 (Ref EPI-ISL_7171744) from Vincent Munster of the NIAID Laboratory of Virology were propagated in TMPRSS2 VeroE6 cells. The clarified culture medium was titrated on Vero E6 hTMPRSS2 cells and the TCID_50_ was determined by the Reed-Muench method implemented in Prism (Graphpad) by Reed Johnson and Nicole Lackemeyer of the NIAID COVID Virology Core Laboratory. Aliquots consisting of 10^2^ to 5 × 10^4^ TCID_50_ of SARS-CoV-2 in 50 µl were administered IN to mice that were lightly sedated with isoflurane. After infection, the weights and morbidity/mortality status were assessed and recorded daily for up to 14 days. The data in each figure are from independent experiments and the same SARS-CoV-2 preparations were used throughout the studies.

### Detection of Wuhan S, Omicron S, and RBD binding IgG and antibodies by ELISA

SARS-CoV-2 (2019-nCoV) spike (S1 + S2 ECD protein, Sino Biologicals), Omicron BA 1.1 spike (from Dr. Raul Cachau, NIAID) or CoV-2 Spike RBD (His-Tag, Genscript) was diluted in phosphate buffered saline (PBS) to a concentration of 1 µg/ml. MaxiSorp 96-well flat-bottom plates (Thermo Fisher) were filled with 100 µl of diluted S protein (0.1 µg/well) and incubated overnight at 4 °C. After adsorption, wells were washed three times with 250 µl PBS + 0.05% Tween-20 (PBS-T, Accurate Chemical). Plates were blocked for 2 h at room temperature with 200 µl PBS-T + 5% nonfat milk and subsequently washed three times with PBS-T. Sera from vaccinated mice were heat-treated at 56 °C for 30 min to inactivate complement and a series of eight 4-fold dilutions of mouse sera were added and the incubation continued for 1 h at room temperature. To detect S-specific IgG antibodies, plates were washed three times with PBS-T and incubated with horse radish peroxidase-conjugated goat anti-mouse IgG (H + L) (Thermo Fisher) for 1 h at room temperature. After incubation plates were washed three times with PBS-T and 100 µl of pre-warmed SureBlue TMB substrate (SeraCare) was added to the plate for 10 min at room temperature. To stop the colorimetric reaction, 100 µl of 1 N sulfuric acid was added to each well and absorbance was measured at A_450_ and A_650_ using a Synergy H1 plate reader with Gen5 analysis software (Agilent Technologies). EC_50_ values were determined using Prism.

### Pseudovirus neutralization assays

BHK-21 cell lines expressing SARS-CoV-2 codon optimized spikes with truncation of the 19 C-terminal amino acids were prepared and used to generate rVSVΔG–GFP-CoV-2 spike pseudoviruses^18^. For the rVSVΔG pseudoviral neutralization assay, serial dilutions of heat-inactivated sera from vaccinated mice were incubated with the amounts of rVSVΔG pseudoviruses that infected 20 to 30% of the cells and a 1:16 dilution of anti-VSV-G I1 hybridoma supernatant (ATCC# CRL-2700) for 45 min at 37 °C. The mixture was then added to VeroE6 cells expressing hTMPRSS2 and hACE2 and incubated for 20 h at 37 °C. The cells were fixed in 2% paraformaldehyde and GFP measured by flow cytometry. NT50 values were calculated using Prism to plot dose-response curves, normalized using the average of the no virus wells as 100% neutralization, and the average of the no serum wells as 0%. The limit of detection (LOD) of 25 was determined by taking 1.96 standard deviation of the mean titer of the control MVA samples.

### Quantitation of infectious SARS-CoV-2

Lungs, brains, and nasal turbinates were homogenized, cleared of debris by centrifugation at 3800 × *g* for 10 min and serial 10-fold dilutions were applied in quadruplicate to Vero E6 hTMPRSS2 cells in DMEM + Glutamax (ThermoFisher) supplemented with 2% heat-inactivated FBS and 1% Antibiotic-Antimycotic in 96-well microtiter plates. After 72 h, the plates were stained with crystal violet and the Reed-Muench method (Prism) was used to determine the concentration at which 50% of the cells displayed a cytopathic effect (TCID_50_).

### Determination of CoV-2 RNA in lungs and nasal turbinates

Following homogenization of the two lungs and turbinates in our BSL-3 facility, RNA was extracted with Trizol Plus RNA Purification Kit with Phasemaker tubes (Thermo Fisher)^12^. The procedure was shown by us to eliminate detectable infectious virus and the RNA was approved for transfer to a BSL-2 facility where contaminating DNA was removed using theTurbo DNA-free kit (Thermo Fisher) and RNA was reverse-transcribed using the iScript cDNA synthesis kit (Bio-Rad, Hercules, CA). CoV-2 S and N transcripts and 18S rRNA were quantified by ddPCR with specific primers (CoV-2 RNA Leader, Forward—CGA TCT CTT GTA GAT CTG TTC TCT AAA C; CoV-2 S, Reverse—TCT TAG TAC CAT TGG TCC CAG AGA; CoV-2 N, Reverse—GGT CTT CCT TGC CAT GTT GAG T; 18 S, Forward—GGC CCT GTA ATT GGA ATG AGT C; 18 S, Reverse—CCA AGA TCC AAC TAC GAG CTT)^12^ using an automated droplet generator and QX200 Droplet Reader (Bio-Rad). The values for CoV-2 S and CoV-2 N sgRNAs were determined relative to the 18S RNA in the same sample.

### Statistics

Measurements were taken on distinct samples and calculations were carried out in Graphpad Prism v. 9 using one-way ANOVA with either Tukey or Dunnetts post-hoc, two-sided Mann-Whitney, two-sided Welch’s t-test or test for linear trend as indicated in Figure Legends or text.

### Safety and ethics

All studies with infectious SARS-CoV-2 were approved by the NIH Institutional Biosafety Committee and carried out in a BSL-3 or ABSL-3 facility. Experiments and procedures involving mice were approved under protocol LVD29E by the NIAID Animal Care and Use Committee according to standards set forth in the NIH guidelines, Animal Welfare Act, and US Federal Law. Euthanasia was carried out using carbon dioxide inhalation in accordance with the American Veterinary Medical Association Guidelines for Euthanasia of Animals (2013 Report of the AVMA Panel of Euthanasia). Experiments with SARS-CoV-2 were carried out under BSL-3 containment.

### Reporting summary

Further information on research design is available in the Nature Research Reporting Summary linked to this article.

## Supplementary information

Supplementary Figures

REPORTING SUMMARY

## Data Availability

All data in the paper and supplemental material are available for use and additional information provided upon request. Biological materials are available upon request with approval of requestors institutional biosafety committee.

## Code availability

No special codes were used.
