# Supplementary material for: Protection from SARS-CoV-2 Variants by MVAs expressing matched or mismatched S administered intranasally to mice
Source: NPJ Vaccines. 2023 Mar 27;8:47. doi: 10.1038/s41541-023-00645-7 (PMC10040904; doi:10.1038/s41541-023-00645-7)
Supplement: Supplementary file 1 — Supplementary Figures [file 41541_2023_645_MOESM1_ESM.pdf]

Pseudovirus S	Changes compared to Reference Wuhan Sequence Genbank# MN908947.3
Wuhan	Codon optimized for human
Beta	L18F, D80A, D215G, del241-243, R246I, K417N, E484K, N501Y, D614G, A701V
Delta	T19R, G142D, del156-157, R158G, L452R, T478K, D614G, P681R, D950N
Omicron	A67V, del69-70, T95I, G142D, del143-145, del211N, L212I, ins214EPE, G339D, S371L, S373P, S375F, K417N, N440K, G446S, S477N, T478K, E484A, Q493R, G496S, Q498R, N501Y, Y505H, T547K, D614G, H655Y, N679K, P681H, N764K, D796Y, N856K, Q954H, N969K, L981F
**CoV challenge S**	**Changes compared to Reference Wuhan Sequence Genbank# MN908947.3**
Wuhan	No changes
Alpha	L5F, del144Y, N501Y, A570D, D614G, P681H, T716I, S982A, D1118H
Beta	L18F, D80A, D215G, del241-243, K417N, E484K, N501Y, D614G, Q677H, R682W, A701V
Delta	T19R, G142D, del156-157, R158G, A222V, L452R, T478K, D614G, P681R, D950N
Omicron	A67V, del69-70, T95I, G142D, del143-145, del211N, L212I, ins214EPE, G339D, S371L, S373P, S375F, K417N, N440K, G446S, S477N, T478K, E484A, Q493R, G496S, Q498R, N501Y, Y505H, T547K, D614G, H655Y, N679K, P681H, N764K, D796Y, N856K, Q954H, N969K, L981F
**rMVA-S**	**Changes compared to Reference Wuhan Sequence Genbank# MN908947.3**
Wuhan	**R682G, R683S, R685S, K986P, V987P, dERRs, 3XFlag**
Alpha	Del69-70, del144Y, N501Y, A570D, D614G, P681H, **R682G, R683S, R685S**, T716I, S982A, **K986P, V987P**, D1118H, **dERRS, 3XFlag**
Beta	L18F, D80A, D215G, del241-243, R246I, K417N, E484K, N501Y, D614G, **R682G, R683S, R685S**, A701V, **K986P, V987P, dERRs, 3XFlag**
Delta	T19R, G142D, del156-157, R158G, K417N, L452R, T478K, D614G, P681R, **R682G, R683S, R685S**, D950N, **K986P, V987P, dERRs, 3XFlag**
Omicron	A67V, del69-70, T95I, G142D, del143-145, del211N, L212I, ins212EPE, G339D, S371L, S373P, S375F, K417N, N440K, G446S, S477N, T478K, E484A, Q493K, G496S, Q498R, N501Y, Y505H, T547K, D614G, H655Y, N679K, P681H, **R682G, R683S, R685S**, N764K, D796Y, N856K, Q954H, N969K, L981F, **K986P, V987P, dERRs, 3XFlag**

Supplementary Figure 1. Comparison of sequences of S in rMVAs, rVSVs and SARS-CoV-2 variants. All sequences are compared to Wuhan Genbank# MN908947.3 and only differences are listed. Amino acids in red are in RBD; amino acids in purple represent modifications in rMVAs for stability of the prefusion form of S, prevent furin cleavage and endoplasmic retrieval, and add a 3 x Flag tag as previously described for Wuhan (Liu, R. K. *et al.* One or two injections of MVA-vectored vaccine shields hACE2 transgenic mice from SARS-CoV-2 upper and lower respiratory tract infection. *Proc. Natl Acad. of Sci.* **118**, doi:10.1073/pnas.2026785118 (2021). Abbreviation: del, deletion.

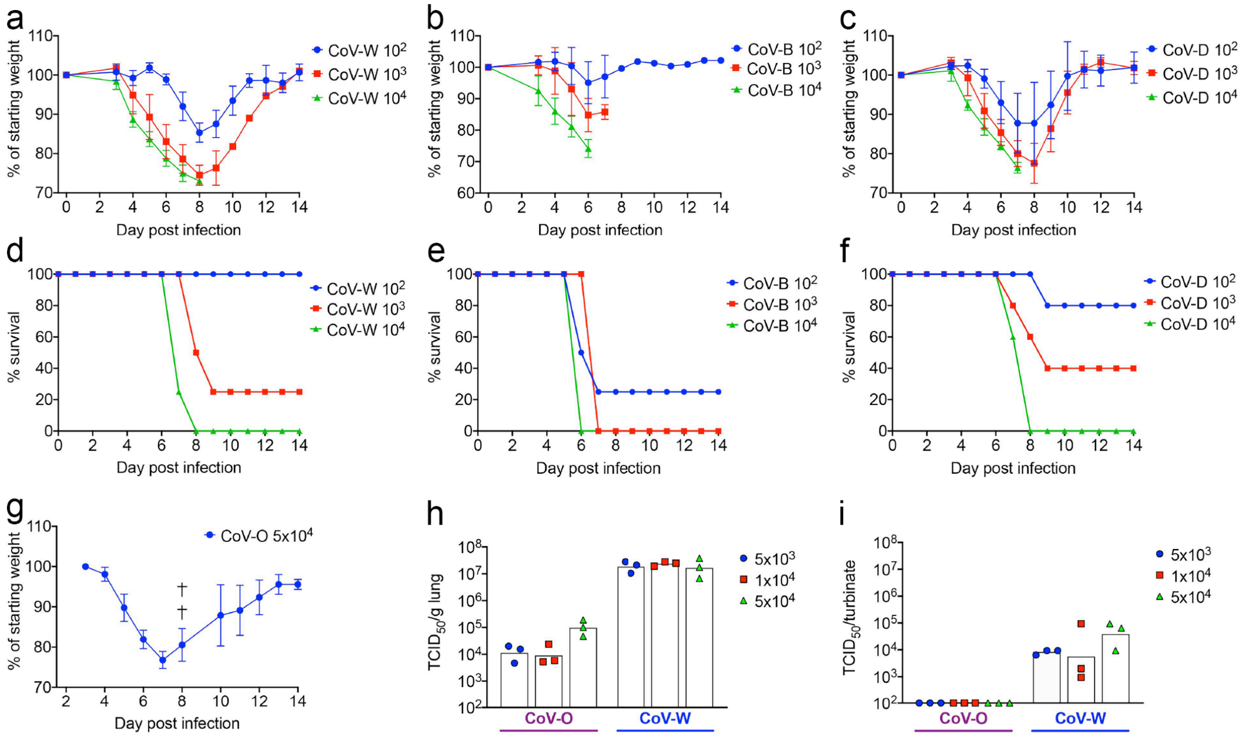

Supplementary Figure 2. **Relative virulence of SAR-CoV-2 variants**. (**a -f**) K18-hACE2 mice (n=3 per group) were infected IN with 10^2 to 10^4 $TCID_{50}$ of indicated SARS-CoV-2 strains Washington (CoV-W, Beta (CoV-B), Delta (CoV-D) and weight loss and survival plotted. **(g)** K18-hACE2 mice (n=5) were infected IN with 5 x 10^4 TCID50 of CoV-O and weight loss plotted. †, death. K18-hACE2 mice (n=3 per group) were infected IN with 5 x 10^3 to 5 x 10^4 $TCID_{50}$ of CoV-W or CoV-O and virus titers in the **(h)** lungs and **(i)** nasal turbinates determined on day 2. Bars are S.D.

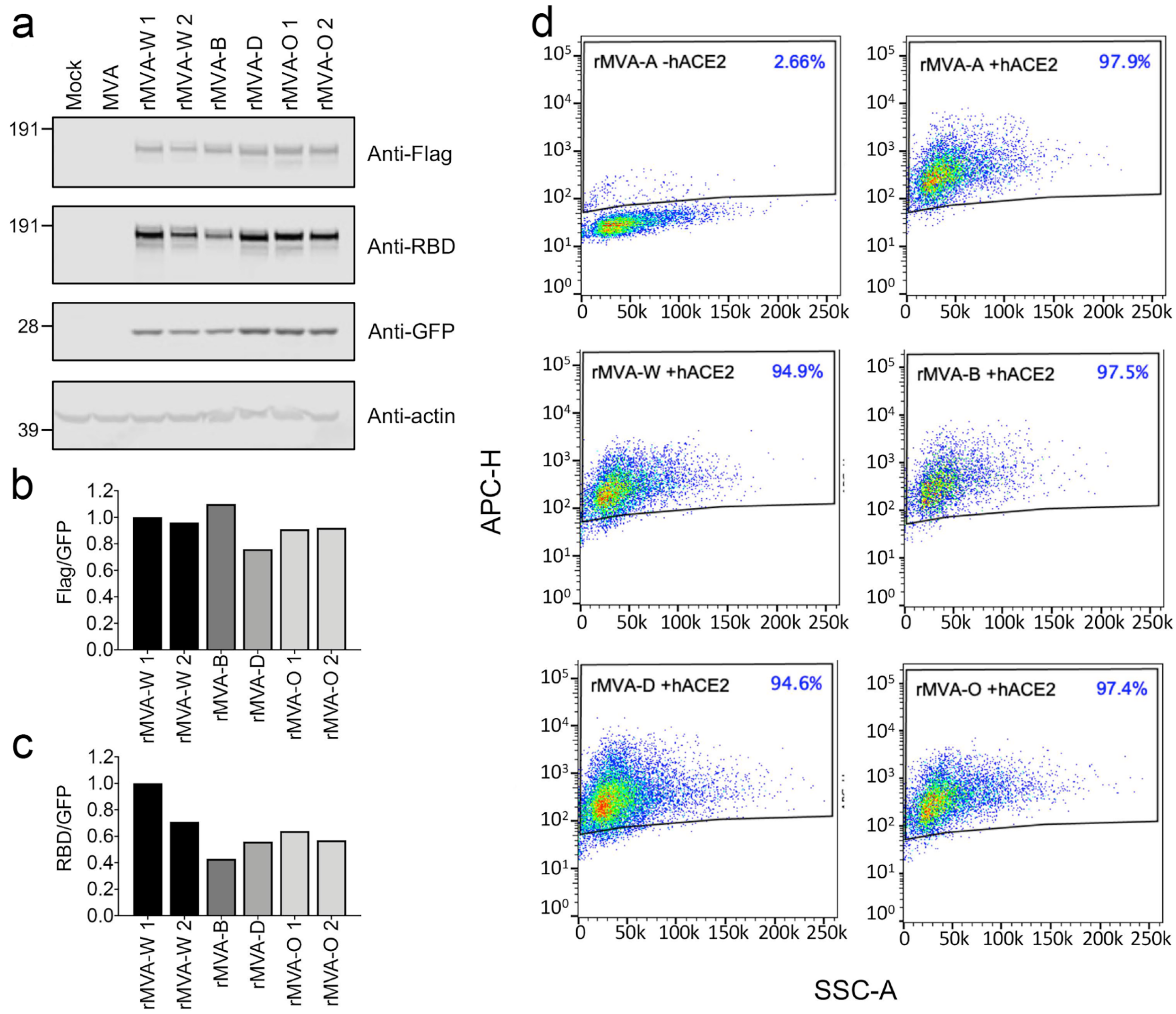

Supplementary Figure 3. Expression of S by variant rMVAs. (a) HeLa cells were infected with 5 PFU per cell of rMVA-W, -B, -D, and -O (two separate clones; O1 was used for other experiments) for 18 h, washed once with phosphate buffered saline, then lysed in LDS sample buffer with reducing agent (ThermoFisher). The lysates were dispersed in a sonicator for four 30 s periods; the proteins were resolved on the same 4 to 12% NuPAGE Bis-Tris gels (ThermoFisher) and transferred to a nitrocellulose membrane with an iBlot2 system (ThermoFisher). The membrane was blocked with 5% nonfat milk in Tris-buffered saline for 1 h, washed with Tris-buffered saline with 0.1% Tween 20, and then incubated at 4°C overnight with a 1:3,000 dilution of rabbit anti-CoV-2 RBD polyclonal antibody (Cat# 40592-T62, Sino Biological) or a 1:5,000 dilution of mouse anti-FLAG M2 antibody (Cat#F1804, MilliporeSigma) in 5% nonfat milk in Tris-buffered saline with 0.1% Tween 20. The membrane was washed and then incubated with a 1:10,000 dilution of IRDye680LT donkey-anti-mouse and IRDye800CW Donkey-anti-Rabbit (LiCor) and imaged on the LiCor Odyssey Imager. The membrane was then probed with a 1:2,000 dilution of mouse anti-actin (Santa Cruz Cat#sc-47778) and a 1:10,000 dilution of rabbit anti-GFP (ThermoFisher Cat# A-11122) and probed with the same secondaries and re-imaged in the LiCor Odyssey. The positions of size markers with mass in kDa are shown on the left. Bands from panel A were quantitated using Odyssey imaging software and the ratios of intensities of the bands probed with **(b)** anti-Flag and **(c)** anti-GFP plotted. **(d)** HeLa cells were infected with 5 PFU per cell of rMVA-A, -W, -B, D and O. The binding of hACE2 to surface expressed S protein on infected HeLa cells was detected by incubating with 100 ng/10^6 cells of biotinylated human ACE2 protein (Cat# 10108-H08H-B, Sino Biological) for 2 h on ice, followed by a 1:200 dilution of APC-Streptavidin (BD Pharmingen Cat#554067) for 30 min on ice in the dark. 5,000 to 10,000 cells were acquired on a FACSCANTO cytometer using Cell Quest software and analyzed with FlowJo (BD Biosciences). Gating was first done on GFP positive cells to identify infected cells,

followed by gating on APC to identify cells expressing spike. The hACE2 was omitted from the upper left panel as a control. Percent of GFP+ cells that stained with hACE2 are indicated.
